# Poly-Infections in a Patient Living With Human Immunodeficiency Virus (HIV)

**DOI:** 10.7759/cureus.103825

**Published:** 2026-02-18

**Authors:** Annarose M Sorvillo, Vadim Belinschi, Camille Akkari, Jihad Slim, Madeline G Ciccone

**Affiliations:** 1 Pharmaceutical Education, Fairleigh Dickinson University College of Pharmacy and Health Sciences, Florham Park, USA; 2 Infectious Diseases, Saint Michael's Medical Center, Newark, USA; 3 Internal Medicine, Saint Michael's Medical Center, Newark, USA

**Keywords:** candida esophagitis, hematologic malignancy, hiv, immunocompromised, neutropenia, strongyloides

## Abstract

Febrile neutropenia (FN) is a medical emergency typically seen in immunocompromised patients with neutrophil counts below 500 cells/µL. It is often associated with chemotherapy, hematologic malignancy, and advanced human immunodeficiency virus (HIV) infection. Severe neutropenia in a low-level viremia and preserved CD4 T-helper cell (CD4) is uncommon and warrants evaluation for alternative etiologies. A 68-year-old man with HIV presented with fever and an absolute neutrophil count (ANC) of zero, without recent chemotherapy or other known myelosuppressive medications. Gram-negative bacteremia was identified, which is often attributed to gut translocation in neutropenic patients. Despite early initiation of tbo-filgrastim, the neutrophil count failed to respond; thus, a bone marrow biopsy was done to evaluate for an underlying marrow disorder. Bone marrow examination combined with genetic and molecular testing revealed a diagnosis of T-cell large granular lymphocytic leukemia (T-LGL), which explained the lack of response to tbo-filgrastim. Because the patient presented with profound neutropenia, gram-negative bacteremia, and a concern of hematologic malignancy, *Strongyloides* serology was ordered even without known travel or residence in endemic areas, and the result was positive. HTLV-1 serology was also obtained due to its known association with *Strongyloides *and gram-negative bacteremia, but it returned negative. This case emphasizes the need to broaden the differential diagnosis for severe neutropenia beyond HIV-related marrow suppression. Undiagnosed T-LGL may present with gram-negative bacteremia and failure to respond to granulocyte colony-stimulating factor (G-CSF), and *Strongyloides* infection should be considered in immunocompromised patients who are being evaluated for occult malignancy, even without identifiable epidemiological risk factors. Early recognition of these conditions can guide timely evaluation and appropriate therapy in complex immunocompromised hosts.

## Introduction

Febrile neutropenia (FN) is defined as a single oral temperature of ≥38.3°C or a sustained temperature of ≥38°C for more than one hour, accompanied by an absolute neutrophil count (ANC) below 500 cells/µL or a count below 1,000 cells/µL with an anticipated decline to ≤500 cells/µL within 48 hours [1,2]. Although classically associated with chemotherapy-induced myelosuppression, FN can also occur in human immunodeficiency virus (HIV) infection; cohort studies have reported rates approaching two-thirds of patients during follow-up, with higher frequency and severity observed in advanced disease [3]. In this population, marrow suppression from viral cytotoxicity, myelotoxic medications, and secondary infections or malignancy contributes to neutropenia. The combination of neutropenia, impaired neutrophil function, and marked CD4+ T-cell depletion substantially increases susceptibility to severe opportunistic infections [3,4].

Gram-negative bloodstream infections (GN-BSIs) remain a major cause of morbidity and mortality, with 30-day mortality rates ranging from 12.1% to 25.3% (*Escherichia coli*:* *12.1%, *Klebsiella *spp*.:* 17.6%, *Enterobacter *spp.: 19.8%,* Proteus* spp.: 20.7%, *Pseudomonas *spp.: 24.7%,* Bacteroides fragilis*: 25.3%) [5]. The growing prevalence of multidrug-resistant gram-negative bacilli, particularly *Escherichia coli*, continues to complicate treatment in neutropenic hosts [5-7]. The current National Comprehensive Cancer Network (NCCN) guidelines recommend prompt initiation of broad-spectrum antibiotics with activity against gram-negative organisms, particularly *Pseudomonas* *aeruginosa*. For high-risk or hospitalized patients, intravenous (IV) monotherapy with an antipseudomonal β-lactam such as cefepime, meropenem, imipenem/cilastatin, or piperacillin/tazobactam is preferred. In carefully selected low-risk patients, oral therapy with ciprofloxacin plus amoxicillin/clavulanate may be considered [1].

*Strongyloides stercoralis* is a soil-transmitted nematode capable of lifelong autoinfection. When immune function is impaired, the parasite can progress to hyperinfection syndrome, leading to widespread larval migration and secondary bacteremia from enteric organisms such as *Escherichia coli.* Concurrent mucosal infections, including *Candida *esophagitis and *Helicobacter pylori* gastritis, may exacerbate gastrointestinal inflammation and facilitate systemic bacterial translocation [8,9]. This case report describes an HIV-positive patient who presented with prolonged neutropenia complicated by *Escherichia coli *bacteremia, *Strongyloides stercoralis *co-infection, possible *Herpes simplex* and *Candida albicans* esophagitis, and *Helicobacter pylori* gastritis. Due to multiple concurrent infections and use of several classes of anti-infective agents, our clinical pharmacist played an invaluable role in providing optimal care to this patient. This case also underscores the critical importance of careful management in patients receiving multiple antimicrobial therapies to minimize drug-drug interactions and ensure continuity of safe, effective treatment during both inpatient and outpatient care.

## Case presentation

A 68-year-old African American man with HIV infection (last known viral load of 60 copies/mL and CD4 count of 400 cells/µL, three months prior to admission; on dolutegravir/rilpivirine), hypertension, bilateral hearing loss, and chronic obstructive pulmonary disease presented with one week of shortness of breath, generalized weakness, myalgias, dysuria, and progressive odynophagia. He also reported subjective fevers and unintentional weight loss. He smokes cigarettes (self-reported six pack-years) and uses heroin via inhalation and intranasal routes. He denied recent travel or known sick contacts. He was born in New Jersey and worked as a mail carrier until retirement.

On presentation, his temperature was 101.2°F, respiratory rate was 18 breaths/minute, heart rate was 103 beats/minute, blood pressure was 115/62 mmHg, and oxygen saturation was 92% on room air. Physical examination revealed bilateral hearing loss with clear ear canals, inspiratory crackles over the left lower lung field, and a regular heart rhythm without murmurs. The remainder of the examination was unremarkable.

**Figure 1:**
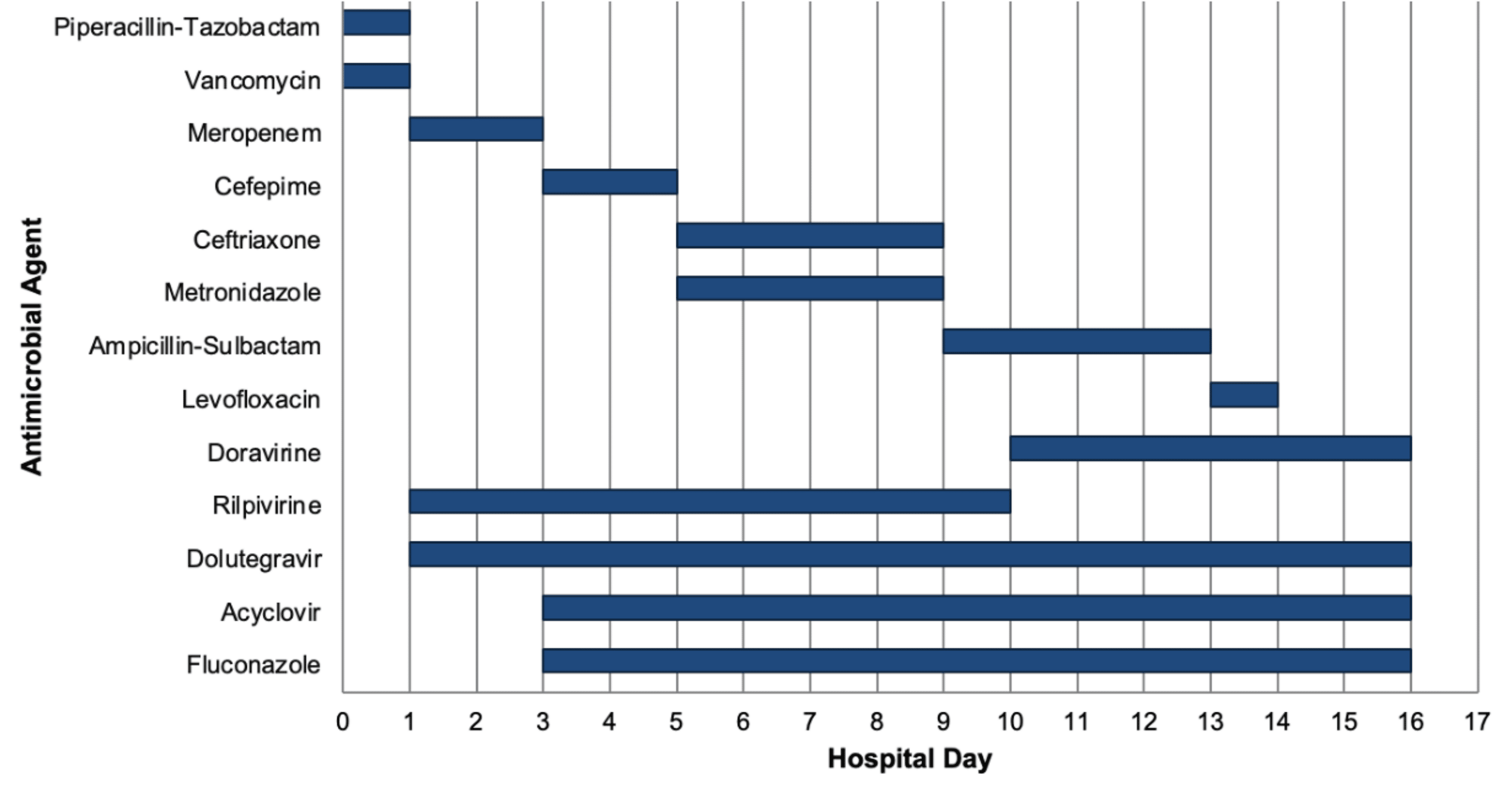
Patient's course of anti-infective agents used during his hospitalization to discharge Hospital days 0-16: Empiric vancomycin and piperacillin-tazobactam were started in the emergency department but discontinued after one dose when preliminary blood cultures showed gram-negative rods, prompting a switch to meropenem. By day 3, the organism was identified as a non-fermenting, non-ESBL *E. coli*. Meropenem was narrowed to cefepime, then to ceftriaxone plus metronidazole for suspected gastrointestinal translocation related to *Strongyloides*. Metronidazole was discontinued when anaerobic coverage was no longer needed. Intravenous ampicillin-sulbactam was selected as step-down therapy based on susceptibility. Once afebrile and able to swallow, he was transitioned to oral levofloxacin. His ART was changed from rilpivirine to doravirine to avoid interactions with the PPI required for *H. pylori* therapy. Based on EGD findings, he was also started on fluconazole for presumed *Candida* esophagitis and acyclovir for suspected HSV esophageal ulcers. ART: antiretroviral therapy, EGD: esophagogastroduodenoscopy, ESBL: extended-spectrum beta-lactamase, HSV: herpes simplex virus, PPI: proton pump inhibitor

Initial laboratory testing demonstrated severe neutropenia with an ANC of zero and mild hyponatremia (Table 1 and Table 2). Computed tomography (CT) of the chest, abdomen, and pelvis showed fecal loading of the ascending and transverse colon and incidental renal cystic changes. CT of the chest showed panacinar emphysema and no infiltrates or consolidation that would be suspicious for pneumonia. He was admitted and started on broad-spectrum antimicrobial therapy, which was later adjusted based on microbiologic results (Figure 1). Tbo-filgrastim (Granix) 300 µg daily was initiated per hematology recommendations. Because of persistent odynophagia in the setting of neutropenia, empiric intravenous (IV) fluconazole and acyclovir were started for suspected *Candida* and herpes simplex virus (HSV) esophagitis.

**Table 1:** Complete blood count within 24 hours of admission

Test	Result	Reference range	Units
White blood cell count	1.9	4.4-11.0	×10³ cells/µL
Red blood cell count	4.99	4.32-5.72	×10⁶ cells/µL
Hemoglobin	10.1	13.5-17.5	g/dL
Hematocrit	32.6	38.8-50.0	%
Mean corpuscular volume	65.3	81.2-95.1	fL
Mean corpuscular hemoglobin	20.3	27.5-33.2	pg
Mean corpuscular hemoglobin concentration	31.1	33.4-35.5	g/dL
Neutrophils (absolute count)	0	1.7-7.0	×10³ cells/µL
Lymphocytes (absolute count)	1.7	0.9-2.9	×10³ cells/µL
Monocytes (absolute count)	0.1	0.3-0.9	×10³ cells/µL
Eosinophils (absolute count)	0	0.0-0.5	×10³ cells/µL

**Table 2:** Complete metabolic panel within 24 hours of admission

Test	Result	Reference range	Units
Sodium	133	136-145	mmol/L
Potassium	3.9	3.5-5.3	mmol/L
Chloride	99	98-110	mmol/L
Carbon dioxide (bicarbonate)	28	20-31	mmol/L
Blood urea nitrogen	33	6-24	mg/dL
Creatinine	0.92	0.70-1.30	mg/dL
Estimated glomerular filtration rate	>90	>90	mL/min/1.73m²
Glucose	106	70-140	mg/dL
Calcium	8.4	8.6-10.4	mg/dL
Anion gap	6	6-19	mmol/L
Albumin	3	3.2-4.8	g/dL
Total bilirubin	0.6	0.2-1.2	mg/dL
Direct bilirubin	0.32	≤0.30	mg/dL
Alkaline phosphatase	41	46-116	U/L
Aspartate aminotransferase	26	10-36	U/L
Lactic acid	1.09	0-2	mmol/L
Procalcitonin	0.58	0.0-0.5	ng/mL
C-reactive protein	14.6	0.0-0.8	ng/mL

**Figure 2:**
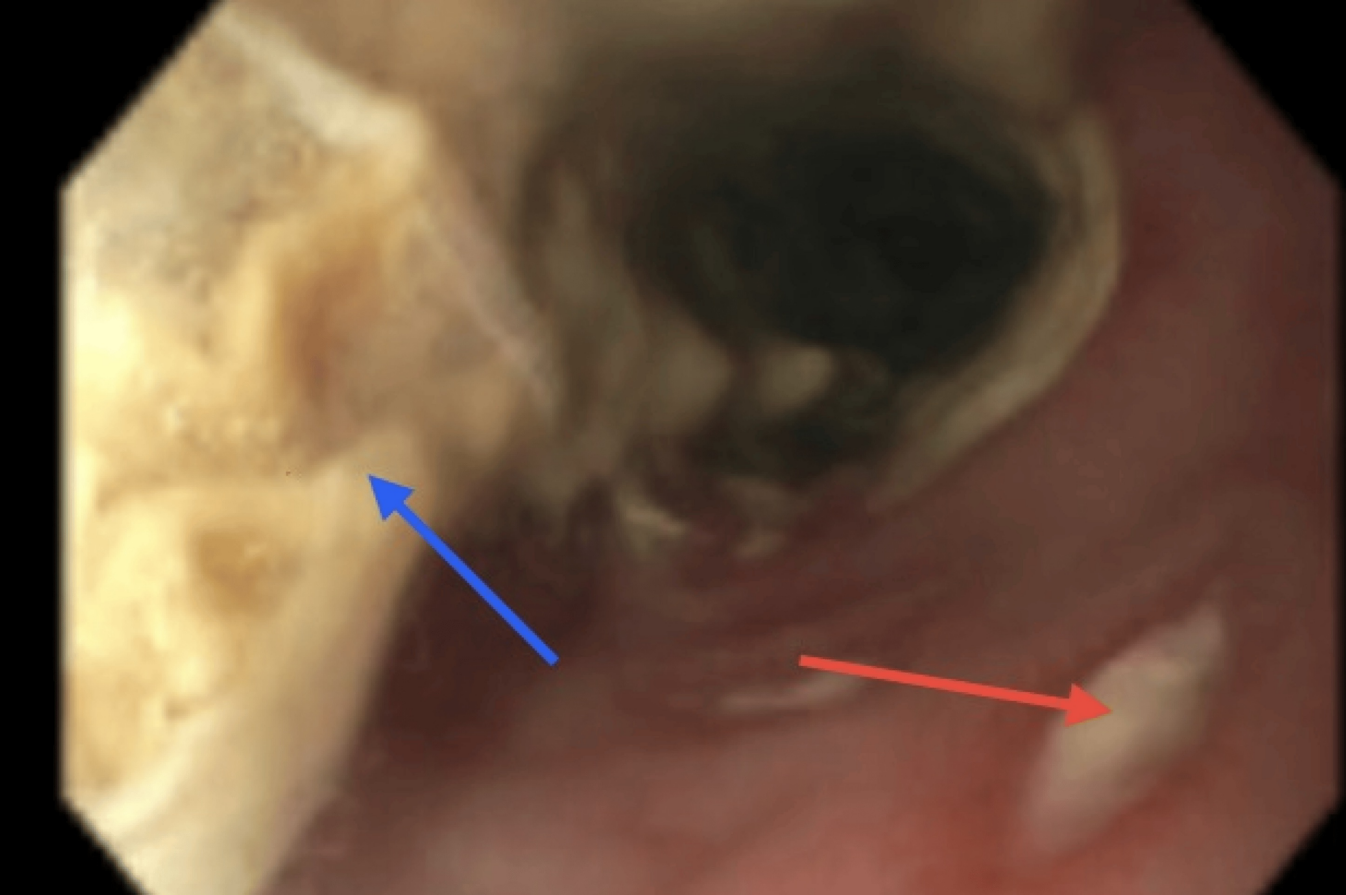
Upper endoscopy images of the proximal esophagus Red arrow: circular crater-like ulcers suspicious for HSV lesions, blue arrow: *Candida *plaques

Esophagogastroduodenoscopy (EGD) revealed white plaques and circular crater-like ulcerations (Figure 2). Pathology confirmed *Candida* species (plaque sample) and *Helicobacter pylori* (*H. pylori*) via immunohistochemistry from the stomach sample. Biopsy of the circular esophageal lesions was negative for herpes simplex virus and cytomegalovirus (CMV). Given concern for sampling limitations and the patient's persistent odynophagia in the setting of profound immunosuppression, acyclovir was continued due to strong clinical suspicion.

After gram-negative bacteremia was identified, gut translocation was initially presumed, as this is the most common source in febrile neutropenia. Urine as a source of gram-negative bacteria was considered; urine analysis was negative for pyuria, and urine culture was negative for growth. Given the severity of his immunosuppression and ongoing evaluation for an underlying hematologic malignancy, *Strongyloides stercoralis* serological testing was pursued despite no known epidemiological exposure, and the result was positive. HTLV-1 serology, obtained due to its known association with *Strongyloides* and hematologic disorders, returned negative.

Despite initiation of tbo-filgrastim, the patient's ANC remained at zero with no early signs of recovery. A bone marrow biopsy showed hypercellular marrow, reduced granulocytic production, and increased T-large granular lymphocytes. Because the patient reported new-onset abdominal pain after multiple doses of granulocyte colony-stimulating factor (G-CSF), splenic enlargement was considered; however, abdominal ultrasound demonstrated no splenomegaly, and further G-CSF administration was discontinued. The patient was treated with two doses of ivermectin for *Strongyloides* infection. His ANC began to recover on hospital day 9, rising from 0 to 0.1. It increased to 0.5 on day 10 and 1.2 on day 11, and reached 3.2 by day 13. He demonstrated clinical improvement with resolution of bacteremia with documented negative culture (on day 4) and a gradual improvement in odynophagia. He was discharged in stable condition with plans for close outpatient hematology follow-up for management of T-LGL.

## Discussion

As the patient’s condition evolved throughout the hospitalization, several important pharmacologic considerations emerged that shaped therapeutic decision-making at each point of care. When initiating therapy for HSV esophagitis, the patient should be started on IV acyclovir, with a transition to oral therapy once lesions begin to improve and continued until complete healing [10]. Although oral therapy was considered, the patient’s odynophagia necessitated extended IV treatment. A key clinical consideration when initiating acyclovir is the need to maintain adequate hydration to prevent crystal nephropathy; however, this was complicated by suspected syndrome of inappropriate antidiuretic hormone secretion (SIADH), characterized by hyponatremia, low serum osmolality, high urine sodium, and a euvolemic physical examination [11]. SIADH and the need for IV acyclovir required a careful balance between meeting the hydration needs of the medication and safeguarding the patient’s electrolyte status, particularly sodium. To overcome this limitation, the patient was given an extended infusion of acyclovir and low-rate fluids.

In a setting where medications were being adjusted in real time, close attention to potential drug-drug interactions remained critical. The patient’s antiretroviral therapy required modification in anticipation of initiating bismuth quadruple therapy (BQT) for *H. pylori* gastritis, given the need to maintain consistent virologic suppression. Rilpivirine depends on an acidic gastric environment for adequate absorption; however, proton pump inhibitors, included in BQT, raise gastric pH and significantly reduce rilpivirine absorption [12]. As a result, rilpivirine was replaced with doravirine, and the patient was discharged on a regimen of dolutegravir and doravirine for the duration of BQT.

Most studies focus on older, treatment-experienced individuals with multimorbidity and polypharmacy, often switched to dolutegravir/doravirine due to drug-drug interactions, cardiovascular/metabolic risk, or resistance to other agents. For example, a multicenter Italian cohort included 157 elderly patients (median age: 59), 75% with multimorbidity and 39% on polypharmacy, with 91% virologically suppressed at baseline. Other cohorts included highly treatment-experienced patients with extensive ART histories and resistance-associated mutations [13-16]. By promptly identifying this interaction before discharging the patient on BQT, the medical team ensured that the HIV regimen remained fully active despite the complexity introduced by concurrent therapies.

Although the patient’s gram-negative bacteremia was most consistent with enteric translocation related to colonic distension and fecal loading, *Strongyloides stercoralis* serology was obtained because he was undergoing evaluation for a possible malignancy. The clinical guidance of the Centers for Disease Control and Prevention recommends screening for chronic* Strongyloides *infection in certain high-risk patients, including those with hematologic malignancy or remote residence in endemic areas. Infection with *Strongyloides* can persist for decades through autoinfection and is typically acquired from soil contaminated with human feces; the absence of recent travel does not reliably exclude prior exposure [17].

*Strongyloides *infection can predispose to bacteremia through mucosal disruption by migrating larvae; the combination of gram-negative rods and positive serology prompted evaluation for HTLV-1/2. HTLV-1 skews immunity toward a Th1-dominant profile, suppressing IL-5 and IgE-mediated eosinophil responses and increasing susceptibility to severe strongyloidiasis [18]. Literature describes translocation of enteric organisms, most commonly *Escherichia coli*, during *Strongyloides *infection, and a recent meta-analysis reports significantly increased odds of both *Strongyloides* infection (OR: ~3.2) and severe disease (OR: ~59.9) in HTLV-1-positive individuals [19]. Although HTLV-1/2 serologies were negative, only screening assays were obtained; confirmatory testing (Western blot, line immunoassay, or proviral DNA PCR) was not performed.

The patient’s severe odynophagia and dysphagia led to EGD, which showed esophageal plaques and ulcerations. Biopsies demonstrated periodic acid-Schiff (PAS)-positive fungal elements and *H. pylori*, while HSV and CMV immunostains were negative. Given sampling limitations and the patient’s clinical improvement on acyclovir, viral esophagitis could not be fully excluded, and empiric antiviral therapy was continued.

Persistent neutropenia despite nine doses of G-CSF prompted hematologic evaluation. Flow cytometry revealed mature T-cell lymphocytosis, an inverted CD4:CD8 ratio, and an elevated population of large granular lymphocytes (24.7%). Bone marrow biopsy confirmed T-cell large granular lymphocytic (T-LGL) leukemia with clonal TCR gene rearrangements. T-LGL leukemia causes cytopenias through chronic cytotoxic T-cell expansion driven by JAK/STAT-mediated survival signaling and, in this patient, manifested predominantly as neutropenia [20]. Cytogenetic and morphologic studies excluded alternative causes such as myelodysplastic syndromes and hemophagocytic lymphohistiocytosis, the latter initially considered due to ferritin > 1,600 ng/mL. Normal splenic imaging ruled out hypersplenism. The patient was ultimately managed with a multidisciplinary approach, with plans for outpatient hematology follow-up and initiation of low-dose methotrexate and prednisone for T-LGL leukemia after completing therapy for opportunistic infections.

## Conclusions

This case highlights the diagnostic and therapeutic complexity that can arise in patients with profound immunosuppression and multiple concurrent infections. The coexistence of *Strongyloides stercoralis*, *Candida albicans*, *Helicobacter pylori*, and *Escherichia coli* bacteremia demonstrates how overlapping infections can obscure the primary source and complicate management. The patient’s neutropenia not only predisposed him to infection but also masked typical inflammatory responses, emphasizing the importance of maintaining a broad differential diagnosis and considering less common etiologies when the infectious source remains unclear. In this context, evaluating for parasitic infections such as *Strongyloides stercoralis* was clinically important, even outside regions of high endemicity, given the unexplained gram-negative bacteremia and the patient’s immunocompromised state. Furthermore, the identification of *Escherichia coli* bacteremia underscored the critical need to maintain a high index of suspicion for enteric translocation in patients with profound, non-responsive neutropenia, even in the absence of primary gastrointestinal symptoms.

From a pharmacologic standpoint, management was additionally complicated by drug-drug interactions between antimicrobial agents and antiretroviral therapy, as well as limitations in fluid administration due to hyponatremia related to the syndrome of inappropriate antidiuretic hormone secretion (SIADH). The need to adjust HIV therapy to accommodate treatment for *H. pylori* highlights the value of multidisciplinary collaboration between infectious disease specialists, pharmacists, and the primary medical team to optimize care.
